# Human Uterine Biopsy: Research Value and Common Pitfalls

**DOI:** 10.1155/2020/9275360

**Published:** 2020-04-28

**Authors:** Alison Maclean, Areege Kamal, Meera Adishesh, Rafah Alnafakh, Nicola Tempest, Dharani K. Hapangama

**Affiliations:** ^1^Department of Women's and Children's Health, Institute of Translational Medicine, University of Liverpool, Member of the Liverpool Health Partnership, Liverpool Women's NHS Foundation Trust, Liverpool L8 7SS, UK; ^2^Pathology Department, Oncology Teaching Hospital, Baghdad Medical City, Baghdad, Iraq; ^3^Lancashire Teaching Hospitals NHS Foundation Trust, Chorley, UK

## Abstract

The human uterus consists of the inner endometrium, the myometrium, and the outer serosa. Knowledge of the function of the uterus in health and disease is relevant to reproduction, fertility, embryology, gynaecology, endocrinology, and oncology. Research performed on uterine biopsies is essential to further the current understanding of human uterine biology. This brief review explores the value of the uterine biopsy in gynaecological and human fertility research and explores the common problems encountered when analysing data generated from different types of uterine biopsies, with the aim of improving the quality, reproducibility, and clinical translatability of future research.

## 1. Introduction

**Figure 1:**
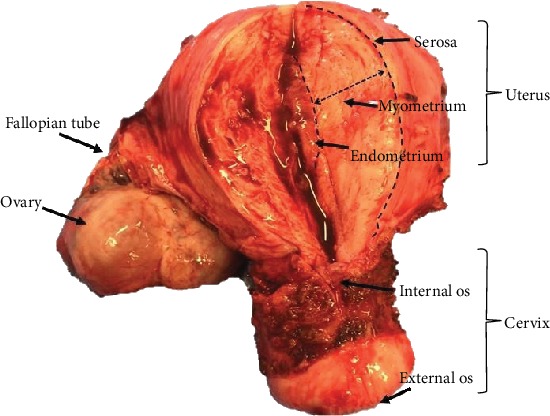
Human uterus and cervix removed at hysterectomy, with a vertical incision on the anterior aspect, representing macroscopic uterine anatomy.

Reproductive, fertility, embryology, and early developmental researchers as well as gynaecologists, endocrinologists, and cancer biologists routinely study the human uterus, as it is an organ pertinent to all of their lines of investigation. The uterus is a hormonally responsive organ, and it is the principal target for many hormones including ovarian sex steroids [1, 2]. The main discerning feature of the human uterus is menstruation, which is a unique process, and is limited to humans and upper-order primates; thus, this particular aspect of human uterine biology cannot easily be modelled in common laboratory animal models (e.g., murine or rodent models) [3]. Therefore, patient-derived uterine biopsies are invaluable to further our current understanding of human uterine biology. The human uterus is a “pear”-shaped pelvic organ with the fundamental purpose of accommodating the embryo/fetus until delivery. The term “uterus” usually excludes the fallopian tubes and the cervix; both of which exist as a continuum of the uterine body. The uterine wall comprises of three layers (Figure 1): (1) the outer serosa which contains the perimetrial cover derived from the embryonic visceral peritoneum; (2) the myometrium containing specialised smooth muscle, stroma, and blood vessels; and (3) the endometrium, which is a complex inner mucosal layer that contains the epithelium, stroma, blood vessels, and leucocytes. The term “uterine biopsy” can therefore represent a tissue sample containing any one or all of these tissue layers. However, the myometrium and endometrium are the two main layers of the human uterine wall that are routinely biopsied and studied by uterine biologists.

## 2. Harvesting the Endometrium

**Figure 2:**
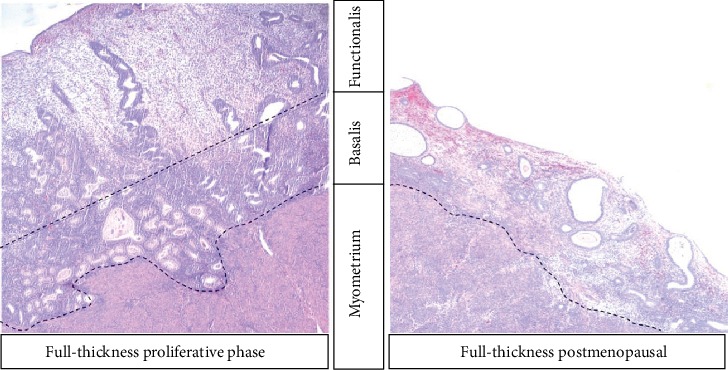
Representative micrograph (4x) of full-thickness human uterine biopsies taken from pre- and postmenopausal women, containing both endometrium and subendometrial myometrium stained with haematoxylin and eosin.

The inner lining of the uterus, the endometrium, is easily accessible through the hollow structure, the cervix, located at the lower part of the uterus, which opens into the vagina. The endometrium extends to the internal cervical os and the upper part of the endocervix (Figure 1). It comprises two functionally distinct layers, the superficial functionalis, and the deeper basalis that lies on the underlying myometrium (Figure 2) [4, 5].

**Figure 3:**
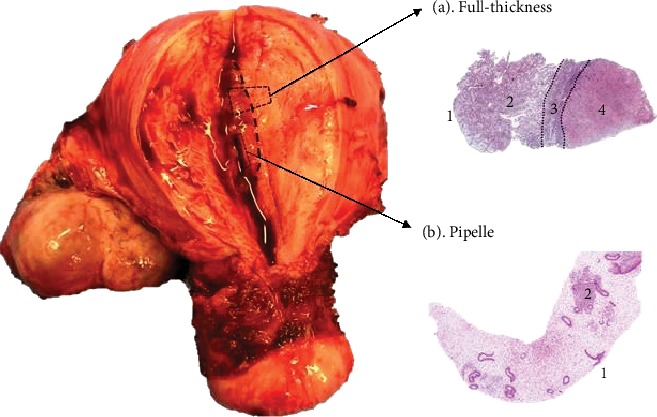
Image of a human uterus with representative micrographs of (a) full-thickness endometrial biopsy (100x) from a hysterectomy specimen and (b) pipelle endometrial biopsy (4x), depicting the distinct anatomical areas included in each of them when the human uterus is sampled with different methods. (1) Luminal epithelium, (2) functionalis, (3) basalis, and (4) myometrium.

In premenopausal women, the functionalis is easily accessible and can be harvested with outpatient and inpatient biopsy methods, either using suction (pipelle) curettes (Figure 3) or scraping with metal curettes. However, the deeper basalis is not usually accessible in a preserved intact premenopausal uterus, and with pipelle or even more extensive curette biopsies, the amount of functionalis obtained depends hugely on the optimal sampling technique and on the cycle phase. For example, pipelle biopsies obtained at the time of spontaneous (menstrual phase) or iatrogenic (traumatic) bleeding may contain mostly blood without much endometrial tissue. Some of these problems can be overcome by adapting special techniques in an intact uterus, such as resection of the endometrium under direct vision with hysteroscopy to obtain a full-thickness endometrial sample containing basalis. However, this is usually an additional and a more invasive procedure, which may not be routinely performed, therefore will require further ethical considerations, necessitating additional time, counselling regarding specific-risks, and thus resources [6]. The premenopausal basalis can also be harvested as a wedge biopsy from a hysterectomy specimen, where a full-thickness endometrial sample can be obtained containing the functionalis, the basalis, and the underlying myometrium [7] (Figure 3). It is important to note, however, that the indication for hysterectomy is usually an underlying uterine pathology, such as heavy menstrual bleeding, endometriosis, or malignancy [8]; therefore, the samples may not be representative of a normal healthy endometrium. Furthermore, most women undergo hysterectomy for benign conditions when medical therapy fails, thus will be routinely still on hormonal treatment, such as the Mirena intrauterine device or gonadotropin-releasing hormone (GnRH) analogue treatments. Such exogenous hormones will alter endometrial phenotype and function, and therefore, a wash out period after hormone exposure is indicated when examining the normal endometrium (of at least 3 months after being on any hormonal therapy) to avoid exogenous hormonal influence. Some authors have harvested placental bed biopsies at the time of delivery of a baby, where decidual tissue with some superficial myometrial tissue is obtained [9].

Contrastingly, in postmenopausal women and premenopausal women on hormonal preparations that preclude the influence of cyclical ovarian hormones on the endometrium, (e.g. GnRH analogues), there is no endometrial functionalis layer. Therefore, when the endometrium is sampled, the harvested biopsies will only contain the stem cell-rich basalis layer of the endometrium [10, 11]. Furthermore, full-thickness biopsies taken from this thin endometrium in a hysterectomy sample (and even possibly from a curettage) invariably contain the underlying myometrium; thus, the studies examining the expression of endometrial-specific genes may be affected by the myometrial expression levels that are included in the whole biopsy. In endometrial cancer research, it is challenging, and not usually possible, to obtain a full-thickness uterine biopsy for snap freezing or RNA studies, as the specimen must be initially preserved whole for pathologists' scrutiny and staging purposes [3]. Hence, curettes or pipelle biopsies are the only available sampling methods for most studies. Depending on the size and the nature of the cancer, a superficial endometrial biopsy may contain a malignant or benign endometrium, or a combination. The actual proportion of cancer cells included in a whole frozen or freshly collected tissue biopsy may affect the data obtained but is not easy to determine. Haematoxylin and eosin (H&E) staining and morphological assessment of the frozen tissue sections may be suitable in some instances. Furthermore, others have also explored the possibility of examining the uterine fluid that represents the cytokine or metabolic milieu of the endometrium related to a particular pathology [12].

There are significant changes in the endometrial cell phenotype and function during different phases of the menstrual cycle [1, 13], including proliferation, decidualisation, and changes in hormone responsiveness. The cellular composition also changes significantly across the menstrual cycle. For example, a sample containing secretory-phase functionalis layer will contain leucocytes accounting for up to 30% stromal cells due to the massive leucocyte infiltration that occurs at the end of the cycle [14]. These changes in cellular proliferation and composition are mainly limited to the functionalis layer, which is relevant to fertility and premenopausal gynaecological conditions, such as heavy menstrual bleeding [15, 16]. Therefore, fertility researchers focus mainly on this layer for the obvious reasons, and they often take biopsies that only contain the functionalis layer from women who wish to preserve their uterus and thus their fertility [17, 18]. In contrast, stem cell biologists focus more on the basalis layer since it has been postulated to harbour endometrial stem cells [5, 10].

Since the endometrium is the primary target organ for ovarian hormones, the endometrial biopsy should naturally be examined in the context of the menstrual cycle phase and thus with relevance to the distinct ovarian hormonal influence of that phase [1]. Frequently, cell type-specific gene expression or protein quantitative studies process whole endometrial samples without necessarily considering the differing cell types included in each sample [19–21]; therefore, cell type-specific changes maybe diluted or masked in the results obtained by analysing the whole biopsy. Unless normalisation using a specific marker is performed, such as marking epithelial cells with cytokeratin to assess the proportion of the particular cell type present in the biopsy, the final results will be prejudiced by the heterogeneity of the cell types included [11, 22, 23].

The cell-cell interaction between the different cell types of the endometrium is also important, with evidence suggesting that cross-talk between the epithelial and stromal cells plays an important role in hormonal regulation, for example, in the context of steroid hormone receptor expression [1, 2]. Methods which do not disturb the tissue architecture but directly assess the gene or protein expression at a cellular level, such as *in situ* hybridisation [11], immunohistochemistry [21], or immunofluorescence [23], will remove this bias. Isolating cells directly can also be achieved with antibody-based fluorescence-activated cell sorting (FACS) [10] or magnetic bead sorting [23] which will also allow extended functional studies in the isolated, viable cells employing either in vitro or in vivo models. Techniques such as laser capture microdissection [24, 25] can also be employed to extract a particular cell type from a frozen tissue section. This method will allow direct comparison and further in-depth analysis of endometrial epithelial and stromal cells from the different endometrial compartments. Furthermore, the novel technique imaging mass cytometry (Fluidigm) has recently been optimised for the first time in the endometrium, broadening our ability to study the endometrium in detail [26]. Techniques like this provide a remarkable platform for highly detailed functional and phenotypic analysis of endometrial tissue, in this case employing metal-labelled antibodies, rather than the fluorochromes that are used in traditional flow cytometry or in immunofluorescence studies. Therefore, it allows simultaneous analysis of a greater number of markers in a single endometrial tissue section. Although cell type-specific spatial expression of genes or gene products can be observed with aforementioned methods, the quantification still poses a challenge. The use of a variety of semiquantitative scoring systems^2^ and computer-based image analysis methods [14] has attempted to reduce observer bias; but none have removed the problem completely. The use of these new techniques and further studies on optimising the analytic methods used will continue to increase our understanding of the human endometrial architecture and cell-specific functions.

In order to avoid common pitfalls, researchers examining endometrial biopsies should follow the already published, elaborative, and standard operating procedures that are available for specific pathologies [7, 27]. These were designed to harmonise the data generated using human endometrial samples and to improve the quality, reproducibility, and clinical translatability of the research.

### 2.1. Solutions for Common Problems with Endometrial Sampling

#### 2.1.1. Pipelle Endometrial Biopsy/Endometrial Curettings

The main problems encountered with these biopsies are (1) the inclusion of insufficient endometrial material and (2) sampling of endometrial functionalis only, unless in certain patients (e.g. postmenopausal women or those on particular hormonal preparations such as gonadotrophin-releasing hormone analogues/progestogens, which curtail the effects of dynamic and cyclical ovarian hormones to produce an endometrial functionalis). To rectify or account for these issues, careful histological evaluation of the biopsy content should be undertaken by a trained histopathologist and reported in the publication accordingly [2, 4, 10, 11, 17].

#### 2.1.2. Harvesting the Endometrium from Hysterectomy Samples

Studies collecting the endometrium from hysterectomy samples should, from the outset, clarify if they intend to collect either full-thickness samples (which requires opening of the uterine cavity) or other scraping or suction methods that would access mostly the endometrial functionalis. When the full-thickness endometrium is collected, the methodology should include details on the exact location in the uterine cavity (e.g., fundal, anterior, or posterior wall) where the biopsy is taken. If the study compared data generated using samples of the endometrium from women who have only a basalis layer (e.g., postmenopausal), with the endometrium of premenopausal women who have a thicker endometrium with obvious basalis and functionalis regions, it is essential to clarify the exact layer that is compared to provide robust and reproducible results [2]. Inclusion of myometrium cells in the whole “endometrial” sample in a full-thickness sample can affect some data. For example, if myometrial cells have a distinct gene expression pattern that may erroneously be interpreted as relevant to the endometrium. The use of specific techniques such as laser capture microdissection would allow accurate isolation of a particular cell type in this instance. If the endometrium is obtained from the hysterectomy specimen using suction or scraping without opening the uterus, the precautions mentioned in Section 2.1.1 above should be followed.

## 3. Harvesting the Myometrium

Myometrial samples are most commonly harvested during a caesarean section (CS), as the myometrium is easily accessible from the uterine incision [28]. The uterine incision at a routine CS is a transverse incision on the anterior aspect of the lower uterine segment. The lower segment of the uterus is typically developed at late gestation and in labour and lies between the physiological retraction ring and the obstetrical internal os [29]. It is a thinner, more fibrous part of the myometrium, which is also less active than the rest of the uterine corpus in a labouring uterus [30, 31]. A myometrial biopsy can be taken from the upper or the lower edge of the transverse uterine incision [28]. Myometrial functional studies as well as many molecular biological studies are often performed on these biopsies. However, the results can be affected by obesity, diabetes, gestation, the presence or absence of labour, singleton or twin pregnancy, and fibrosis from previous uterine surgery [32–35]. Furthermore, data should be interpreted in the context of these biopsies being obtained from a gravid uterus, which has undergone massive hypertrophy [36] and has been exposed to the pregnancy related hormonal milieu [37]. The location of the myometrial biopsy may also have implications for data analysis; for example, a biopsy taken from the upper uterine segment at a classical CS (where a vertical incision is made into the upper uterine segment) may have different properties to the myometrium obtained from a lower uterine segment [38]; however, in relation to contractility studies, current evidence suggests that there is no significant difference between myometrial biopsies obtained from upper and lower uterine segments [39].

The nonpregnant myometrium can be sampled at the time of gynaecological surgery, typically after hysterectomy [40] or at myomectomy. Myomectomy samples may contain leiomyomatous tissue, but a sample obtained at hysterectomy can be taken from any part of the uterus, with or without commonly occurring pathological lesions such as leiomyomata or adenomyomas [16]. Interestingly, published functional studies commonly compare the nonpregnant and pregnant myometrium, for example assessing the contractility of muscle fibres, but do not necessarily consider the impact of comparing muscle fibres derived from different uterine areas [41, 42]. This may impact the quality of the data generated, for example, if the non-pregnant myometrial fibres were obtained from the uterine fundus, whilst pregnant myometrium was derived from the lower segment of the uterus [43]. This issue is further complicated by the fact that no discernible lower segment exists in a nonpregnant uterus; thus, we propose that attempts should be made to collect myometrial tissue from the anterior lower uterine body in nonpregnant uterus and from the upper edge of the transverse CS incision to strive to obtain similar tissue for comparative studies. Finally, since the myometrium contains distinct myometrial layers [43, 44], the exact layer muscle fibres are derived from may also confound the results.

## 4. Conclusion

In summary, uterine biopsies are a valuable source of human tissue, and studies using these biospecimens have contributed significantly to our current understanding of pregnancy, fertility, and many gynaecological disorders. When collecting and processing uterine biopsies for studies and biobanking, careful consideration is essential of the many pitfalls that can confound the results obtained from them, and strict adherence to published standard operative procedures is also imperative. This will enable researchers to utilise these important biospecimens to discover diagnostic and therapeutic targets for the benefit of millions of women suffering from obstetric and gynaecological conditions.
